# A Pan-Cancer Analysis Reveals the Prognostic and Immunotherapeutic Value of Stanniocalcin-2 (STC2)

**DOI:** 10.3389/fgene.2022.927046

**Published:** 2022-07-22

**Authors:** Zhong-Hui Jiang, Xianfeng Shen, Yanhong Wei, Yongji Chen, Hongbo Chai, Lingyun Xia, Weidong Leng

**Affiliations:** Department of Stomatology, Taihe Hospital, Hubei University of Medicine, Shiyan, China

**Keywords:** stanniocalcin-2, head and neck squamous cell carcinoma, prognostic biomarkers, target therapy, pan-cancer

## Abstract

**Background:** Stanniocalcin-2 (STC2) is a secreted glycoprotein which plays an important role in regulating the homeostasis of calcium, glucose homeostasis, and phosphorus metastasis. Accumulating evidence suggests that STC2 is implicated in cancer mechanisms. However, the effects of STC2 on cancer development and progression across pan-cancer are not yet completely known.

**Methods:** Data were downloaded from The Cancer Genome Atlas database to obtain differentially expressed genes significantly associated with prognosis (key genes). A gene was selected for subsequent correlation studies by integrating the significance of prognosis and the time-dependent ROC curve. Gene expression of different tumor types was analyzed based on the UCSC XENA website. Furthermore, our study investigated the correlation of STC2 expression between prognosis, immune cell infiltration, immune checkpoint genes (ICGs), mismatch repair genes (MMRs), tumor mutation burden (TMB), microsatellite instability (MSI), and drug sensitivity in various malignant tumors. Gene set enrichment analysis (GSEA) was conducted for correlated genes of STC2 to explore potential mechanisms.

**Results:** A total of 3,429 differentially expressed genes and 397 prognosis-related genes were identified from the TCGA database. Twenty-six key genes were found by crossing the former and the latter, and the highest risk gene, STC2, was selected for subsequent correlation studies. STC2 had good diagnostic performance for HNSCC, and was closely related to the survival status and clinicopathological stage of HNSCC patients. In pan-cancer analysis, STC2 was upregulated in 20 cancers and downregulated in seven cancers. STC2 overexpression was overall negatively correlated with overall survival, disease-free survival, disease-specific survival, and progress-free survival. STC2 was profoundly correlated with the tumor immune microenvironment, including immune cell infiltration, ICGs, MMRs, TMB, and MSI. Moreover, STC2 was significantly negatively correlated with the sensitivity or resistance of multiple drugs.

**Conclusion:** STC2 was a potential prognostic biomarker for pan-cancer and a new immunotherapy target.

## Introduction

Head and neck squamous cell carcinoma (HNSCC) is the sixth most common cancer, and the 5-year overall survival rate of HNSCC is only 40–50% by incidence worldwide (Bray et al., 2018). Every year, approximately 600,000 HNSCC cases are diagnosed worldwide (Cohen et al., 2016). HNSCC arises in mucosal epithelium including oral cavity, pharynx, and larynx and is tightly linked to exposure to tobacco-derived carcinogens, alcohol consumption, and human-papillomavirus (HPV) infection (Nakagawa et al., 2021). The prognosis for patients with HNSCC is largely determined by tumor stage at presentation. Early-stage tumors have a favorable prognosis, whereas advanced cancer has poor prognosis. Clinically, most HNSCC patients are diagnosed with advanced cancer with lymph node metastasis. Therefore, this creates an urgent need for practical biomarkers to help in accurate early diagnosis and prediction of tumor prognosis.

Stanniocalcin-2 (STC2) is a secreted glycoprotein which plays a pivotal role in regulating the homeostasis of calcium, glucose homeostasis, and phosphorus metastasis (Takei et al., 2012). STC2 has been shown to play a regulatory role in various tissues and organs in an autocrine or paracrine manner (Chen and Zhu 2008). The correlation between STC2 and tumor progression has been investigated over the last 5 years in multiple studies. STC2 is a useful molecular marker predicting the progression and prognosis of digestive system tumors, including hepatocellular carcinoma (Wang et al., 2019), pancreatic cancer (Lin et al., 2019), gastric cancer (Ke et al., 2019), and colorectal cancer (Zhang et al., 2019). STC2 has also affected extrahepatic cholangiocarcinoma (Li and Yang 2020), nasopharyngeal carcinoma (He et al., 2019), laryngeal cancer (Ding et al., 2021), and osteosarcoma (Yang et al., 2021). STC2 activates ITGB2/FAK/SOX6 and PI3K–AKT signaling pathways in some cancers (Yang et al., 2017; Li et al., 2022). On the other hand, several studies showed that STC2 is a protective factor for some tumors. STC2 reduces resistance to sunitinib in clear-cell renal cell carcinoma (Qin et al., 2022). STC2 inhibits migration and invasion of breast cancer *via* inhibition of PKC signaling (Hou et al., 1932). Therefore, it is necessary to perform a pan-cancer analysis to reveal the role and underlying molecular mechanisms of STC2 in clinical phenotypic characteristics and tumor immune microenvironments of multiple cancers.

In the present study, we first integrated the samples from the UCSC XENA website to identify the differentially expressed genes (DEGs) and prognosis-related genes. We further screened key genes by crossing the DEGs and prognosis-related genes and selected the highest risk key gene for subsequent pan-cancer analysis. We investigated the correlation between the gene expression and prognosis, immune microenvironment, immune checkpoint genes (ICGs), tumor mutation burden (TMB), microsatellite instability (MSI), mismatch repair genes (MMRs), and immune/stromal scores.

## Materials and Methods

### The Datasets

The RNA sequencing (RNA-seq) data from The Cancer Genome Atlas (TCGA) database and the genotype–tissue expression (GTEx) project were downloaded from the UCSC XENA website (https://xenabrowser.net/datapages/). These data included the 33 different tumor types and matched normal tissues from TCGA and 31 normal human tissues from GTEx. Corresponding clinical profiles were downloaded from these two databases. Duplicated data from these two data sources were excluded. The appropriate gene expression profiles were downloaded from the gene expression omnibus (GEO, http://www.ncbi.nlm.nih.gov/geo/) to test the results of the TCGA data analysis.

### Functional Enrichment Analysis of Upregulated Differentially Expressed Genes

The enrichment of gene ontology (GO) in terms of upregulated DEGs and the Kyoto Encyclopedia of Genes and Genomes (KEGG) pathways was analyzed using the Goplot and ggplot2 package of R. P-value <0.05 was considered significant. GO is a tool widely used for exploring the potential functions of targets, including molecular functions, biological pathways, and cellular components.

### Identification of Prognosis-Related Differentially Expressed Genes

The DEGs were identified by “Limma” package of R software (version 3.6.3). The results were filtered using adjusted *p*-value < 0.05 and |log_2_^(fold change)^| > 2 as the threshold to eliminate the potential false-positive results from analysis of TCGA or GTEx. The volcano plot and heat map were plotted using the R package “ggplot2.” In addition, the HNSCC patients were split into a high expression group and a low expression group based on median expression levels of genes. The potential prognostic genes that related to overall survival (OS) in HNSCC were screened using the univariate Cox analysis. The potential prognosis-related DEGs (key genes) were obtained using the R package “VennDiagram.” Time-dependent receiver operating characteristics (ROC) curves for 1, 3, and 5 years were plotted to estimate the predictive power of the key genes. The significance of prognosis and time-dependent ROC curve was further integrated to identify a key gene for follow-up research.

### Analysis of the Significance of Stanniocalcin-2 Expression in Prognosis of Head and Neck Squamous Cell Carcinoma and Pan-Caner

The expression level of STC2 between normal tissue and tumor tissues in HNSCC patients was evaluated. To investigate the diagnostic performance of STC2, the ROC curve was plotted and the area under the curve (AUC) was calculated. An evaluation of the correlation between STC2 expression level and clinicopathological parameters (e.g., survival status, clinical stage, histologic grade, and T stage) was performed by Pearson’s chi-square test. In addition, we further combined the GTEx database to explore the expression level of STC2 in 33 cancers and corresponding normal tissues to offset the deficiency of a small sample size of normal non-cancerous tissue in TCGA. The Human Protein Atlas (HPA) (https://www.proteinatlas.org/) database was used to illustrate the STC2 protein distribution among normal and cancer tissues. HPA is one of the world’s most frequently visited biological databases. It can provide researchers with high-quality immunohistochemistry images of proteins in the tissue atlas and pathology atlas panels (Digre and Lindskog 2021).

### Survival Analysis

The association of STC2 with OS, disease-free survival (DFS), disease-specific survival (DSS), and progress-free survival (PFS) was evaluated using Cox regression in various cancer types. According to expression levels of STC2, the HNSCC patients in TCGA were divided into two groups (high and low expression levels) based on the median expression level of STC2 as a cutoff. The analysis process was realized *via* “survival” and “survminer” packages of R software. The results were visualized using the “forest plot” R package.

### Immunological Correlation Analysis

To evaluate the association between STC2 expression level and tumor microenvironment (TME) in 33 tumors, the immune scores and stromal scores of each sample were analyzed *via* the R package “ESTIMATE.” The relationship between STC2 expression and immune checkpoint genes in the TME was examined *via* the online platform “SangerBox” (http://sangerbox.com/Tool). *p*-value < 0.05 was considered statistically significant.

### Correlation Analysis of Stanniocalcin-2 With Tumor Mutation Burden, Microsatellite Instability, and Mismatch Repair Genes

TMB, MSI, and MMRs have been proved to be important biomarkers of the TME. The data of five MMRs (EPCAM, PMS2, MLH1, MSH2, and MSH6) and MSI were obtained from the TCGA database. The correlation between STC2 expression levels and MMR gene expression levels, MSI, and TMB was evaluated using the Pearson correlation coefficient. Differences with a *p*-value < 0.05 were considered to be statistically significant.

### Gene Set Enrichment Analysis

GSEA is a computational method that determines whether an *a priori* defined set of genes shows statistically significant, concordant differences between two biological states. The samples were divided into two groups (high- and low-expression groups) based on the STC2 expression levels. KEGG and HALLMARK pathways in the high- and low-expression groups were analyzed using GSEA. The functions involved in both groups were yielded by setting a random combination of 1,000 analyses. A normalized enrichment score (NES) was calculated.

### Drug Sensitivity Analysis

To clarify the influence of STC2 expression levels on drug sensitivity and drug tolerance, the processed gene expression and drug sensitivity data were downloaded from the CellMiner database (https://discover.nci.nih.gov/cellminer/). Drugs without FDA approval or clinical trials were removed. The correlation was analyzed using Pearson’s correlation coefficient test, and the cut-off *p*-value was set to 0.05. The results were visualized using the “limma” and “ggplot2” packages.

### Genetic Alteration Analysis

To clarify basic characteristics of STC2, the GeneCards database (https://www.genecards.org/) and COMPARTMENTS (https://compartments.jensenlab.org/Search) database were used to find the genomic location and subcellular location of STC2. The cBioPortal web (https://www.cbioportal.org/) was further utilized to explore the genetic alteration of STC2. All TCGA tumor samples were selected and STC2 mutation rates were queried in different cancers by entering “STC2” in the “TCGA firehose legacy studies.” In addition, a three-dimensional structure diagram of STC2 was obtained.

## Results

### Differentially Expressed Genes in Head and Neck Squamous Cell Carcinoma

**FIGURE 1:**
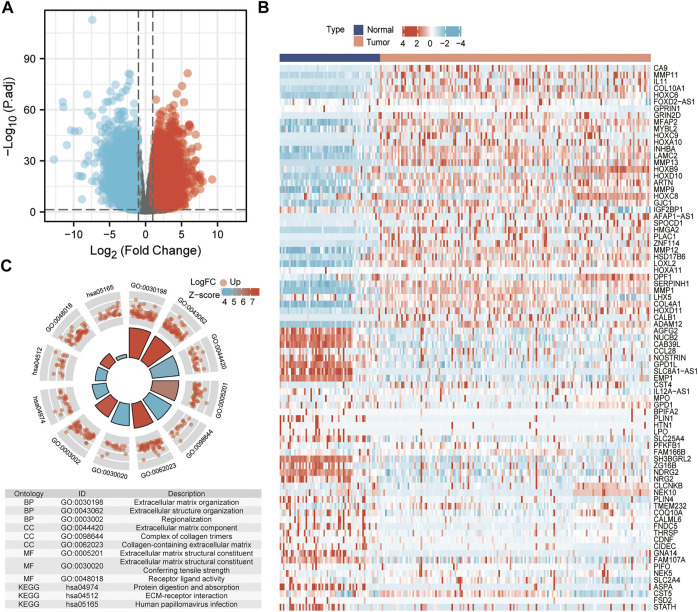
Identified differentially expressed genes. **(A)** Volcano plot: up-regulated and down-regulated genes were indicated in red dots and blue dots, respectively. **(B)** Heatmap of the differentially expressed genes. **(C)** GO terms and KEGG pathways enrichment analysis.

According to the threshold which set adjusted *p*-value < 0.05 and |log_2_^(fold change)^| > 2, a total of 3,429 DEGs (1,861 up-regulated genes and 1568 down-regulated) were identified, as visualized by volcano plots (Figure 1A). To verify the results, an expression profile (GSE30784) of 229 sample data was downloaded from the GEO database. The top 50 most up- and down-regulated genes were plotted in a heat map based on the sample RNA-seq data in GSE30784 (Figure 1B). The results indicated that the screening result of the differential analysis was stable and reliable in the present study. We used GO and KEGG functional enrichment analysis to annotate the function of DEGs (Figure 1C). It was found that the genes significantly upregulated were associated with structural roles for cells.

### Prognosis-Related Genes and Their Prognostic Significance for Head and Neck Squamous Cell Carcinoma

**FIGURE 2:**
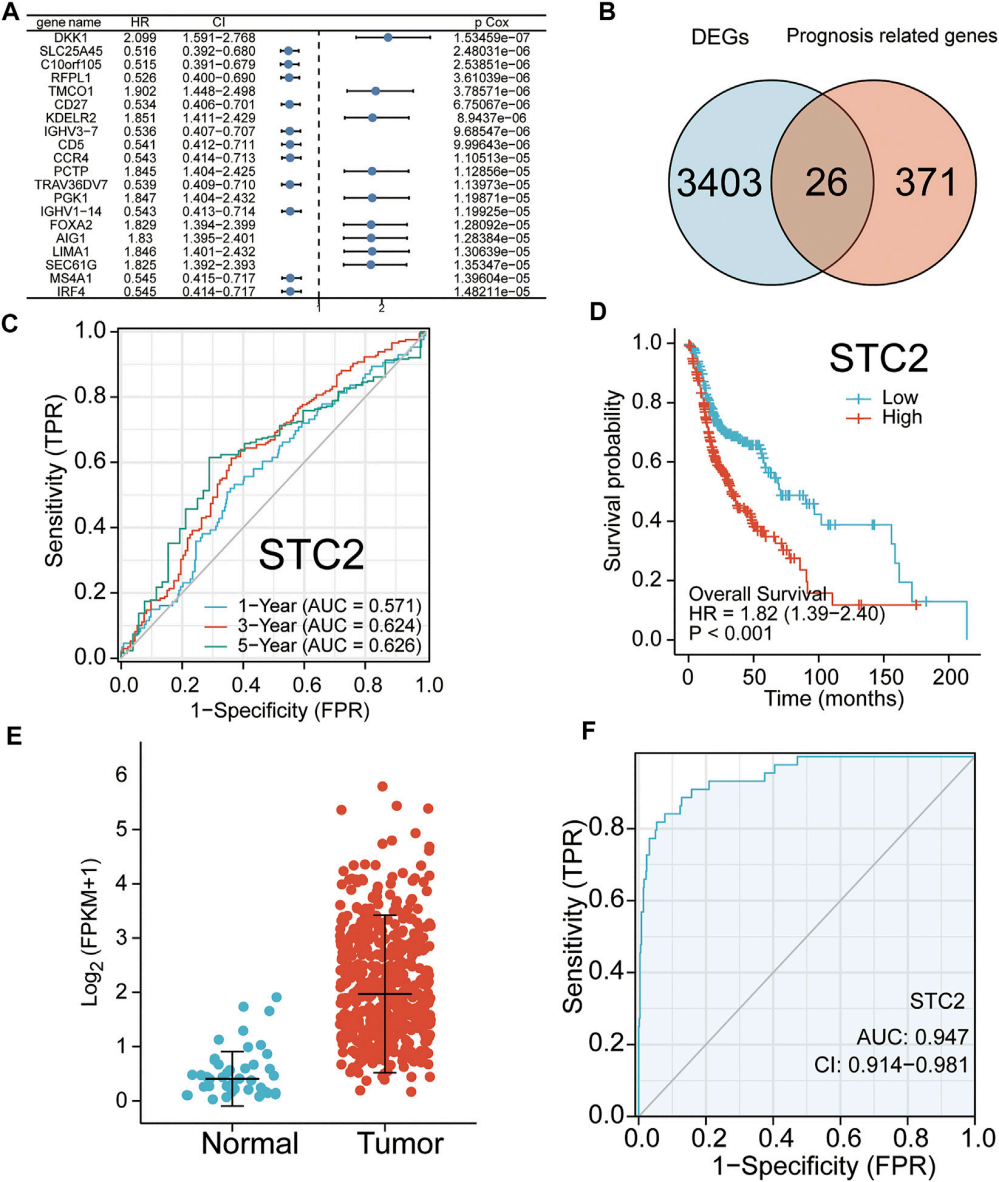
Identified key genes and their examined prognostic significance. **(A)** Top 20 genes with the highest correlation to overall survival (OS) are shown. **(B)** Crossing the 3,429 DEGs with 397 prognosis genes. **(C)** Kaplan–Meier curve of STC2. **(D)** Time-dependent receiver operating characteristics (ROC) curves of STC2 for 1, 3, and 5 years **(E)** STC2 mRNA levels in HNSCC tissues (*n* = 502) and normal tissues (*n* = 44). **(F)** Diagnostic significance of STC2 in HNSCC. ROC curves were plotted and the area under the curve (AUC) of STC2 was 94.7%.

**FIGURE 3:**
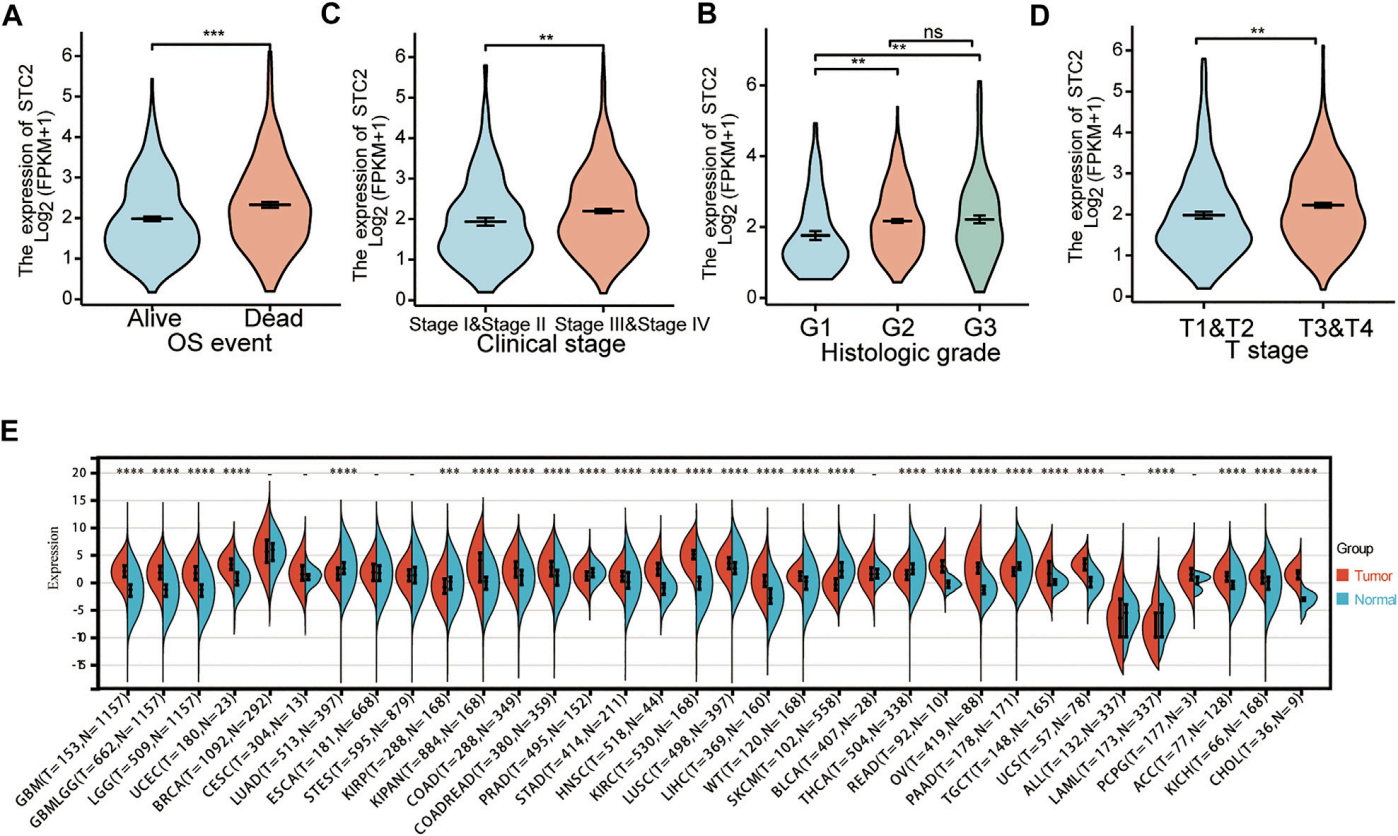
Analysis of the correlation between STC2 expression and clinicopathology. **(A)** Expression levels of STC2 in different survival status of HNSCC. **(B)** Expression levels of STC2 in early and advanced cancer. **(C)** Expression levels of STC2 in different degrees of tumor differentiation. **(D)** Expression levels of STC2 in different tumor stages. **(E)** Expression levels of STC2 in 33 cancer types. *p < 0.05; **p < 0.01; ***p < 0.001; ****p < 0.0001.

A total of 397 prognosis-related genes were identified based on the univariate Cox analysis (*p* < 0.001). The top 26 genes with the largest significant difference were shown in the forest plot (Figure 2A). The key genes were filtered by crossing the 3,429 DEGs with the 397 prognosis-related genes. The screening result is shown using a Venn plot (Figure 2B). The time-dependent ROC curve of all key genes is shown in Figure 2C and Supplementary Figure S1. Integrating the significance of prognosis and time-dependent ROC curve, the mRNA STC2 was selected for subsequent studies. The survival curve of STC2 is shown in Figure 2D. Our study analyzed the expression of STC2 in HNSCC. Compared with normal tissues (*n* = 44), the STC2 expression level was significantly increased in cancerous tissues (*n* = 502) (Figure 2E). The ROC curves confirmed that upregulation of STC2 played a significant performance in the efficacy of HNSCC diagnosis (AUC = 0.947) (Figure 2F). Furthermore, the present study analyzed the correlation of STC2 expression with clinicopathology. The results showed that the STC2 expression level was significantly up-regulated in the HNSCC patients of dead, stage III–IV, G2–G3, and T3–T4 (Figures 3A–D).

### The Expression of Stanniocalcin-2 in Pan-Cancer

**FIGURE 4:**
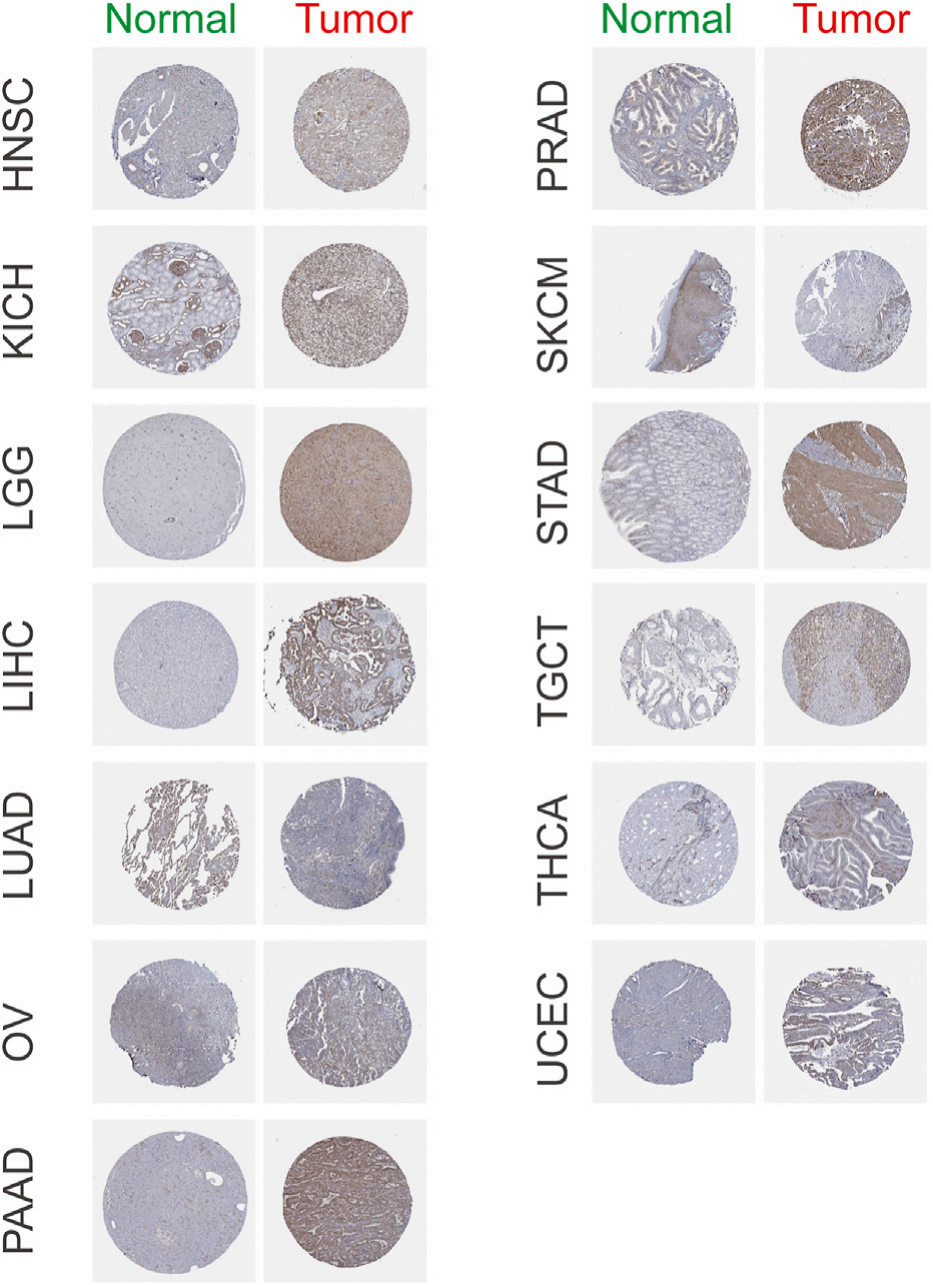
Representative immunohistochemistry images of STC2 in HNSC, KICH, LGG, LICH, LUAD, OV, PAAD, PRAD, SKCM, STAD, TGCT, THCA, and UCEC based on The Human Protein Atlas.

The STC2 expression levels were analyzed in multiple tumors from the TCGA and GTEx databases. The results showed that the expression level of STC2 was significantly different among 27 tumors, except breast invasive carcinoma (BRCA), cervical squamous cell carcinoma and endocervical adenocarcinoma (CESC), esophageal carcinoma (ESCA), stomach and esophageal carcinoma (STES), bladder urothelial carcinoma (BLCA), acute lymphoblastic leukemia (ALL), and pheochromocytoma and paraganglioma (PCPG) (Figure 3E). Using the HPA database, we found that the expression level of STC2 was significantly increased in HNSC, LGG, LIHC, OV, PAAD, PRAD, STAD, TGCT, THCA, and UCEC. However, STC2 expression was significantly decreased in LUAD and SKCM (Figure 4).

### Analysis of Association of Stanniocalcin-2 Expression With Survival in Pan-Cancer

**FIGURE 5:**
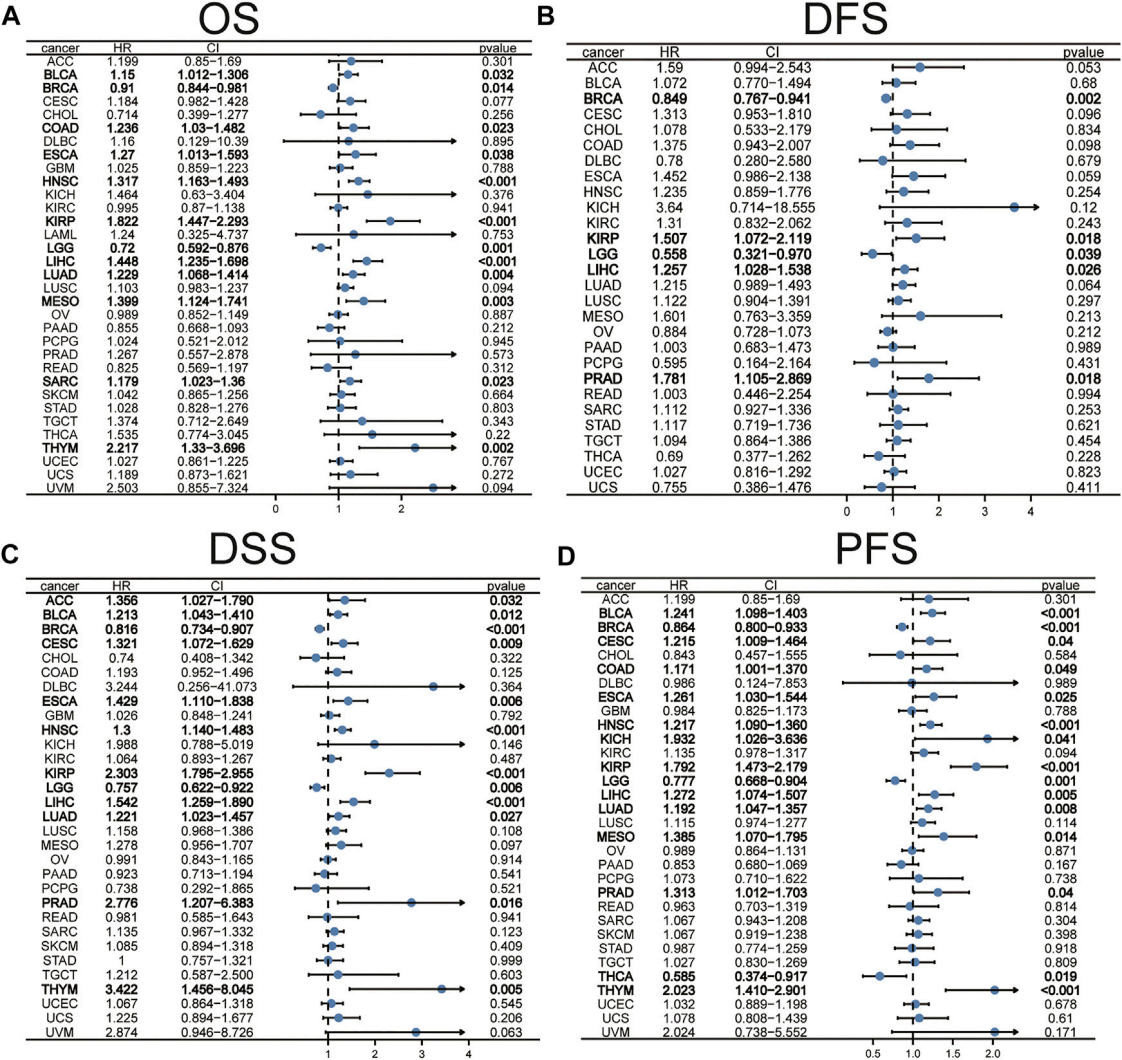
The prognostic significance of STC2 in pan-cancer. The associations between STC2 expression levels and overall survival **(A)**, disease-free survival **(B)**, disease-specific survival **(C)**, and progress-free survival **(D)** in various cancer types were illustrated by the forest plots.

The prognostic significance of STC2 in different cancers was evaluated using Cox regression analyses of the TCGA database. The results showed that high expression levels of STC2 were associated with poorer OS in BLCA, COAD, ESCA, HNSC, kidney renal papillary cell carcinoma (KIRP), liver hepatocellular carcinoma (LIHC), lung adenocarcinoma (LUAD), mesothelioma (MESO), sarcoma (SARC), and thymoma (THYM), but predicted better prognosis in BRCA and brain lower-grade glioma (LGG) (Figure 5A). The correlation analysis of disease-free survival (DFS) found that the STC2 expression level was significantly associated with the prognosis of BRCA, KIRP, LGG, LIHC, and PRAD (Figure 5B). The DSS analysis revealed that high expression of STC2 was significantly associated with poor prognosis in 10 tumors and improved prognosis in two tumors (Figure 5C). According to data on PFS, the high expression of STC2 was significantly associated with prognosis in 12 out of 32 tumors (Figure 5D).

### Analysis of the Correlation Between Stanniocalcin-2 and Immunity

**FIGURE 6:**
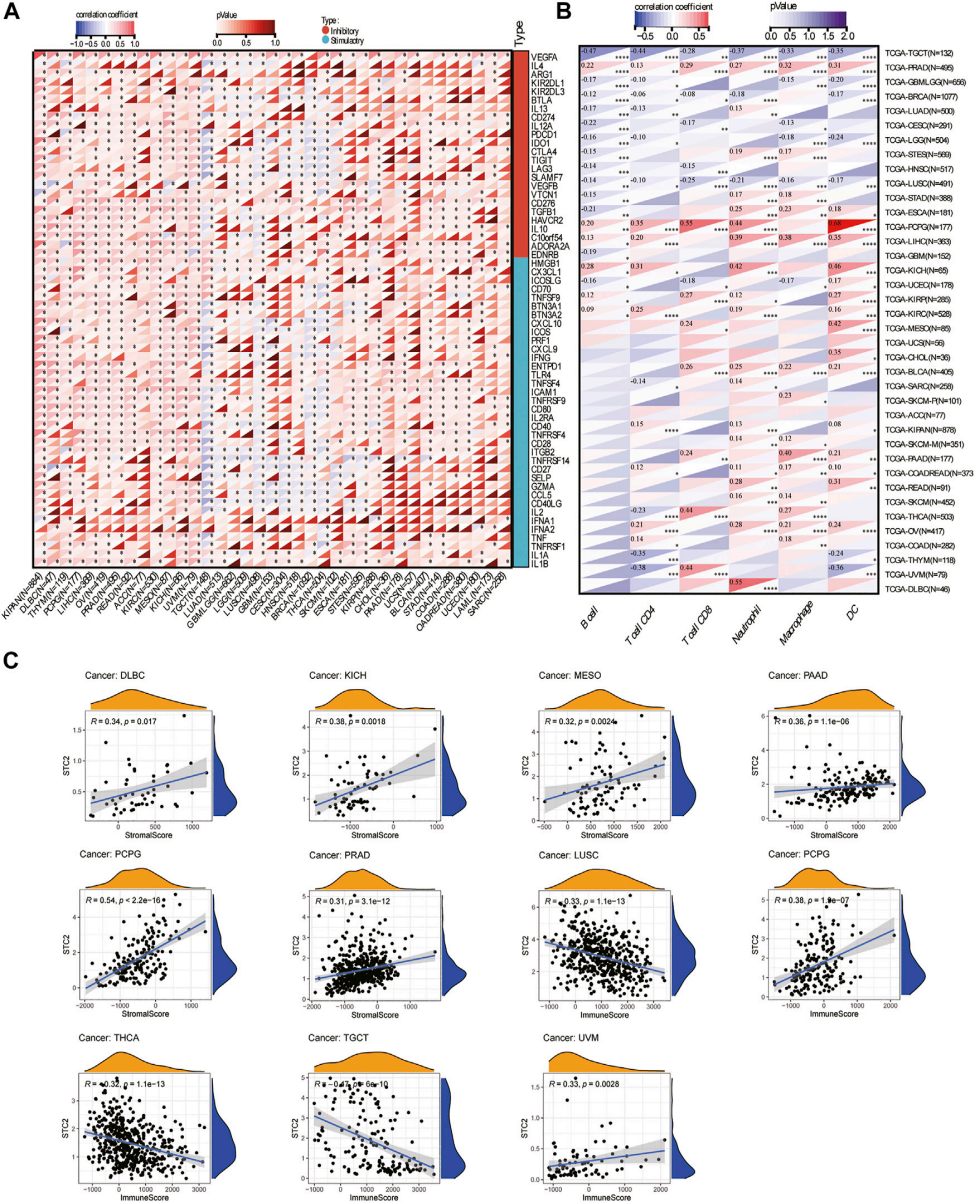
Immunological correlation analysis. **(A)** The correlation between STC2 expression and immune checkpoint genes. **(B)** Correlation of STC2 expression with immune cell infiltration in 33 cancers. Blue represents negative correlation and red represents positive correlation. *p < 0.05; **p < 0.01; ***p < 0.001; ****p < 0.0001. **(C)** Correlation between STC2 and stromal scores and immune score.

To evaluate the role of STC2 in the tumor microenvironment, we analyzed the relationship between STC2 expression level and 60 common ICP gene expressions, including 24 inhibitory and 36 stimulatory. The results showed that correlation between STC2 and ICP genes varied across tumor types (Figure 6A). STC2 expression level was positively correlated with 60 ICP genes in most tumors, including HNSC, BLCA, acute myeloid leukemia (LAML), stomach adenocarcinoma (STAD), and uterine carcinosarcoma (UCS). However, STC2 expression levels were inversely correlated with 60 ICP genes in testicular germ cell tumors (TGCT). It is suggested that STC2 might be involved in the tumor immune microenvironment by coordinating the activity of specific ICP genes. Our study further explored the association of STC2 with immune cell infiltration in 33 tumors. Immune infiltration analysis showed that the expression level of STC2 was associated with the level of immune cell infiltration (including B cell, CD4^+^ T cells, CD8^+^ T cells, neutrophils, macrophages, and dendritic cells) (Figure 6B). Especially in PCPG, kidney chromophobe (KICH), LIHC, there was a strongly positive correlation between the expression level of STC2 and the immune cell infiltration level. In addition, the immune score and stromal score were calculated by the ESTIMATE algorithm (Figure 6C). The results showed that the STC2 expression levels was significantly correlated with immune score in lymphoid neoplasm diffuse large B-cell lymphoma (DLBC) (r = 0.34, *p* = 0.017), KICH (r = 0.38, *p* = 0.0018), MESO (r = 0.32, *p* = 0.0024), pancreatic adenocarcinoma (PAAD) (r = 0.36, *p* < 0.001), pheochromocytoma and paraganglioma (PCPG) (r = 0.54, *p* < 0.001), and PRAD (r = 0.31, *p* < 0.001). The STC2 expression level was significantly correlated with stromal score in LUSC (r = 0.33, *p* < 0.001), PCPG (r = 0.38, *p* < 0.001), thyroid carcinoma (THCA) (r = 0.32, *p* < 0.001), TGCT (r = 0.47, *p* < 0.001), and UVM (r = 0.33, *p* = 0.0028).

### Analyses of Correlation Between Stanniocalcin-2 Expression Levels and Mismatch Repair Genes, Tumor Mutation Burden, and Microsatellite Instability

**FIGURE 7:**
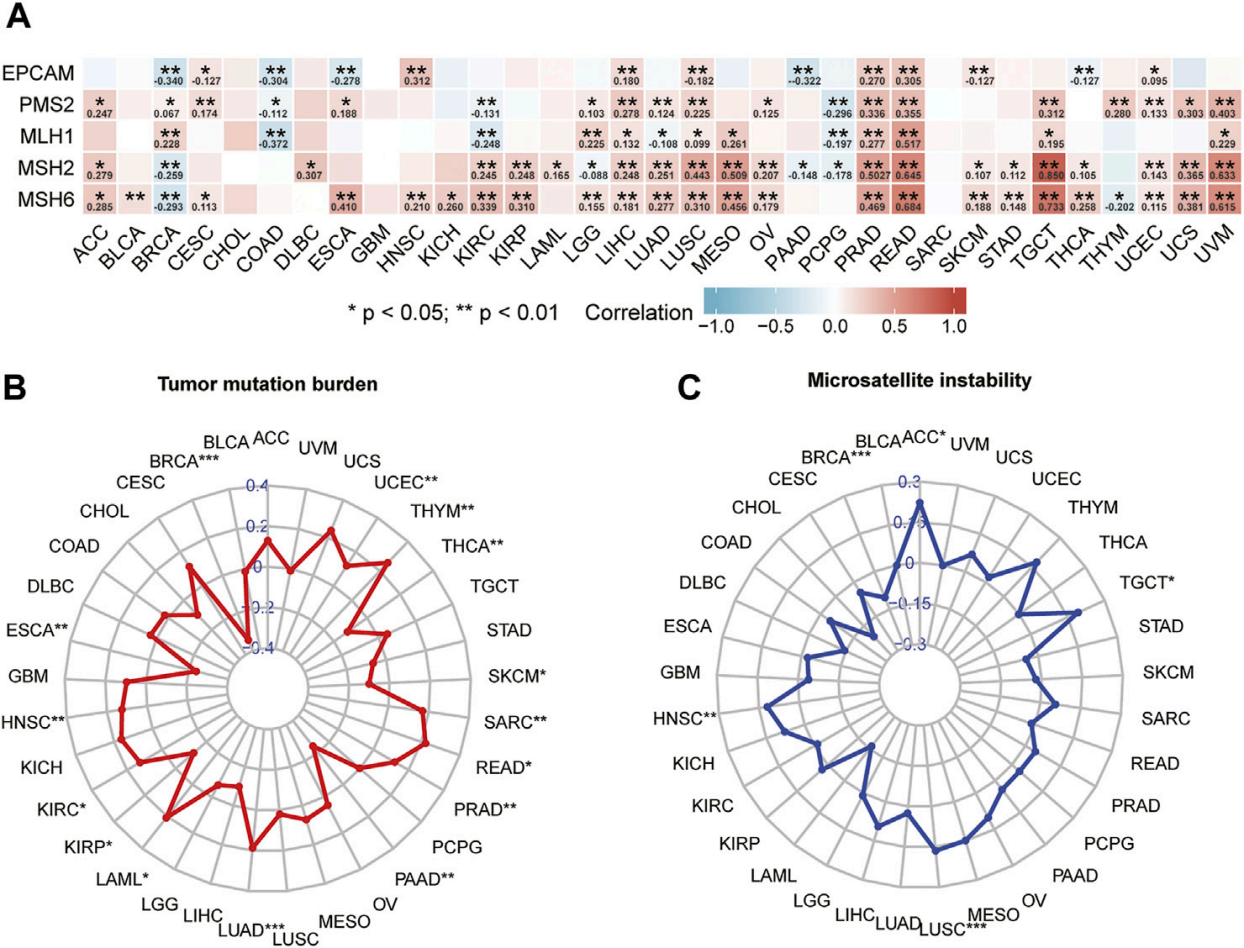
Analysis of correlation between STC2 and mismatch repair genes **(A)**, tumor mutation burden **(B)**, and microsatellite instability **(C)**.

The results of correlation analysis of STC2 expression level with five MMRs are shown in Figure 7A. The results indicated that expression level of STC2 was significantly correlated with MMRs in almost all cancers except BLCA, cholangiocarcinoma (CHOL), DLBC, glioblastoma multiforme (GBM), KICH, LAML, and SARC. The correlation was most significant in BRCA, CESC, LIHC, LUSC, PRAD, and rectum adenocarcinoma (READ). Our study further investigated the relationship between STC2 expression and TMB. The association between STC2 and TMB was significant in 15 cancer types (Figure 7B). Specifically, the significant and positive correlation between STC2 expression level and TMB was found in HNSC (r = 0.13, *p* < 0.01), LAML (r = 0.21, *p* < 0.05), KIRC (r = 0.13, *p* < 0.05), LUAD (r = 0.19, *p* < 0.001), PRAD (r = 0.12, *p* < 0.01), READ (r = 0.22, *p* < 0.05), SARC (r = 0.17, *p* < 0.05), THYM (r = 0.26, *p* < 0.01), and UCEC (r = 0.12, *p* < 0.01). Conversely, STC2 expression levels were negatively correlated with TMB in BRCA (r = −0.34, *p* < 0.001), ESCA(r = 0.24, *p* < 0.01), KIRP (r = −0.12, *p* < 0.05), PAAD (r = −0.24, *p* < 0.01), skin cutaneous melanoma (SKCM) (r = −0.10, *p* < 0.05), and THCA (r = −0.12, *p* < 0.01). In another five types of adrenocortical carcinoma (ACC), BRCA, HNSC, LUSC, and TGCT, STC2 expression was related to MSI (Figure 7C). The results demonstrated that upregulated STC2 expression was significantly associated with increased TMB in ACC (r = 0.22, *p* < 0.05), HNSC (r = 0.12, *p* < 0.01), LUSC (r = 0.17, *p* < 0.001), and TGCT (r = 0.19, *p* < 0.05), while it was associated with decreased TMB in BRCA (r = 0.11, *p* < 0.001).

### Potential Functions of the Stanniocalcin-2–Correlated Genes

**FIGURE 8:**
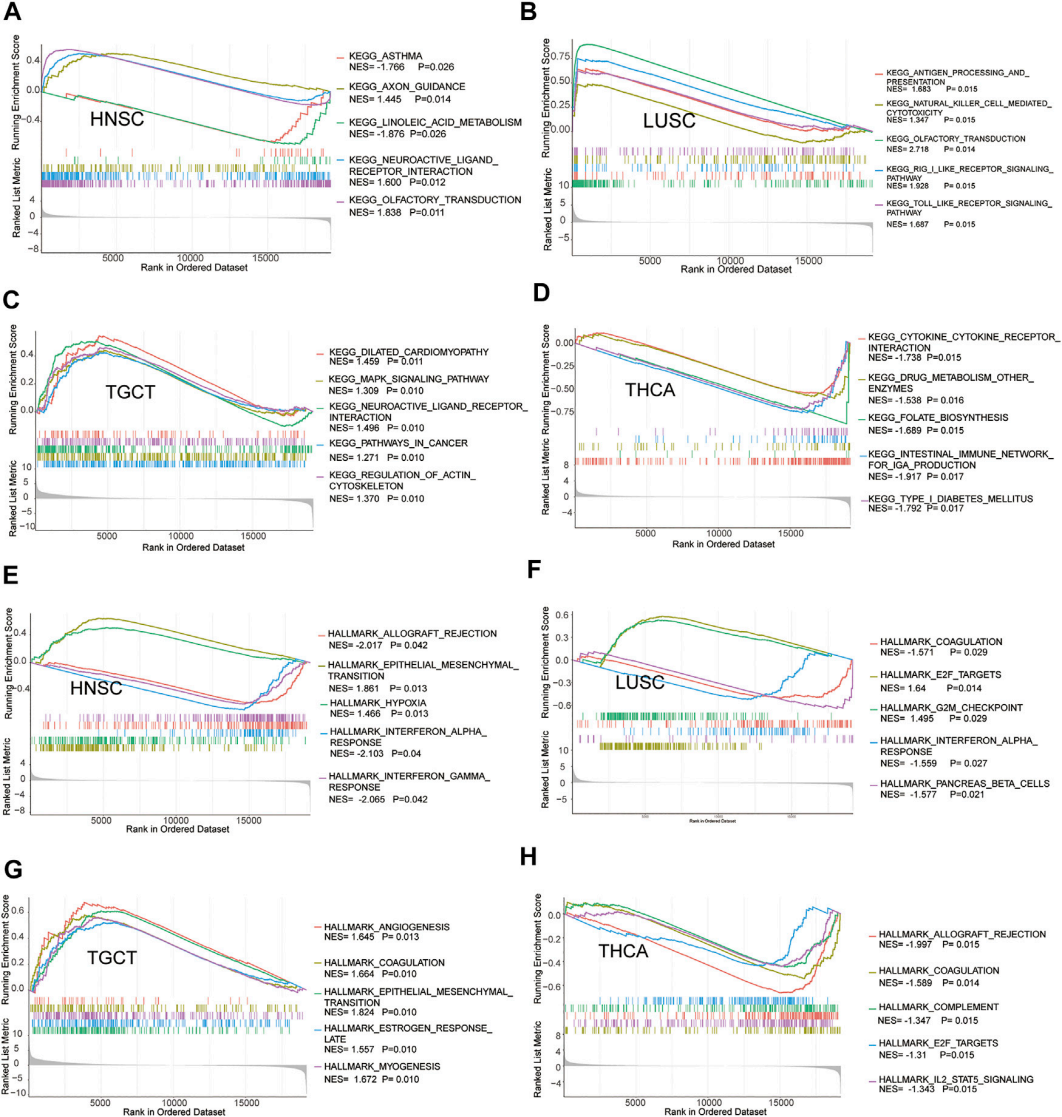
Gene set enrichment analysis of STC2. Enrichments of KEGG pathways in HNSC **(A)**, LUSC **(B)**, TGCT **(C)**, and THCA **(D)**. Enrichments of HALLMARK pathways in HNSC **(E)**, LUSC **(F)**, TGCT **(G)**, and THCA **(H)**.

The GSEA enrichment analysis revealed that STC2 was the focus of many signaling pathways associated with cancer. The KEGG pathway enrichment analysis of upregulated STC2 revealed a significant enrichment of linoleic acid metabolism, olfactory transduction, RIG-I–like receptor signaling pathway, and intestinal immune network for IgA production (Figures 8A–D). The HALLMARK pathway analysis revealed that allograft rejection, epithelial–mesenchymal, interferon α response, and interferon γ response were enriched in high STC2 (Figures 8E–H).

### Drug Sensitivity Analysis

**FIGURE 9:**
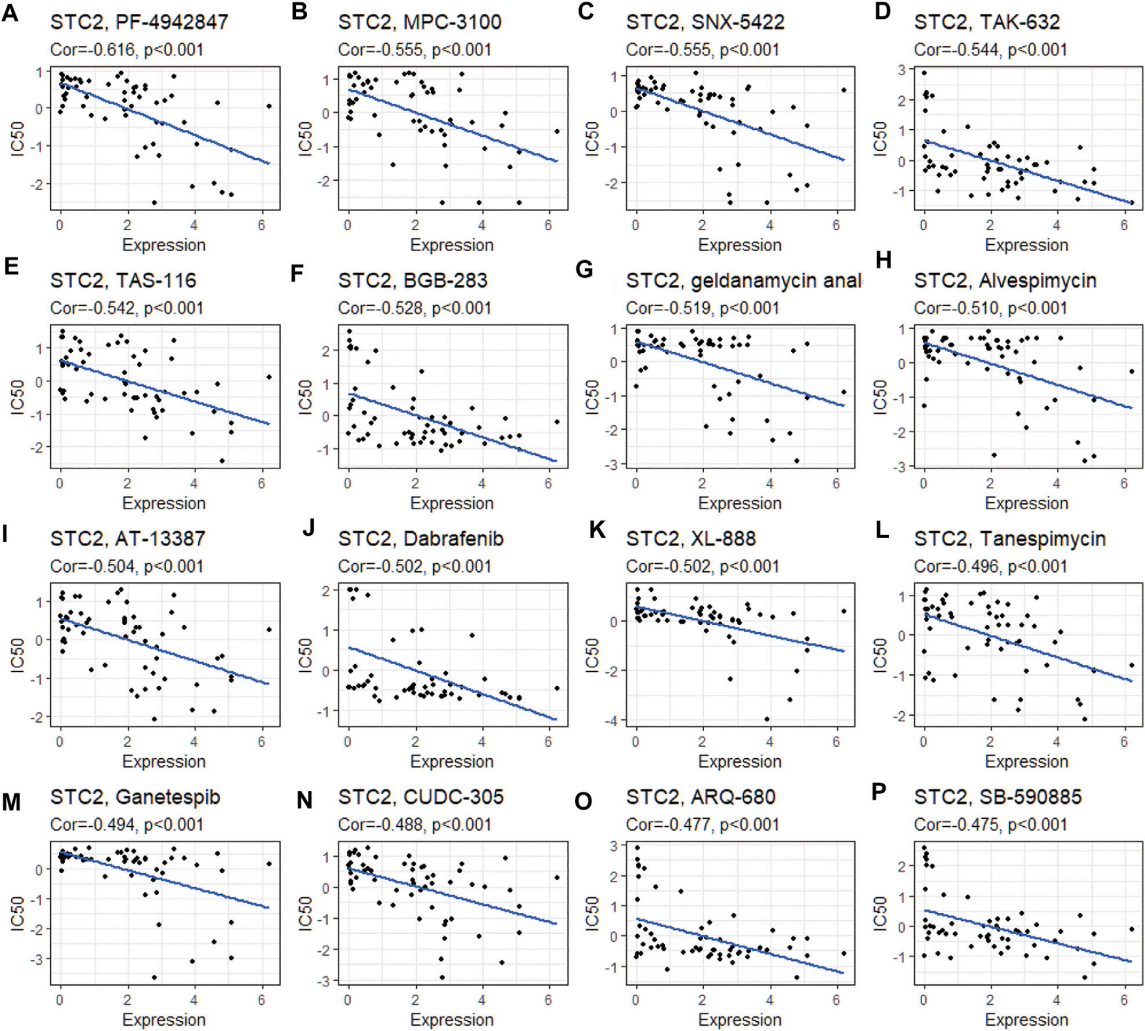
Drug sensitivity analysis of STC2. The expression of STC2 was associated with the drug sensitivity of PF-4942847 **(A)**, MPC-3100 **(B)**, SNX-5422 **(C)**, TAK-632 **(D)**, TAS-116 **(E)**, BGB-283**(F)**, geldanamycin analog **(G)**, alvespimycin **(H)**, AT-13387 **(I)**, dabrafenib **(J)**, XL-888 **(K)**, tanespimycin **(L)**, ganetespib **(M)**, CUDC-305 **(N)**, ARQ-680 **(O)**, and SB-590885 **(P)**.

We analyzed the correlation between STC2 expression and drug sensitivity based on the CellMiner database. The top 16 drugs with the strongest correlations were shown in Figure 9. The results suggested that upregulation of STC2 expression reduced the sensitivity to a number of drugs, including PF-4942847, MPC-3100, SNX-5422, TAK-632, TAS-116, BGB-283, geldanamycin analog, alvespimycin, AT-13387, dabrafenib, XL-888, tanespimycin, ganetespib, CUDC-305, ARQ-680, and SB-590885.

### Basic Biology of Stanniocalcin-2 and Genetic Alteration Analysis

**FIGURE 10:**
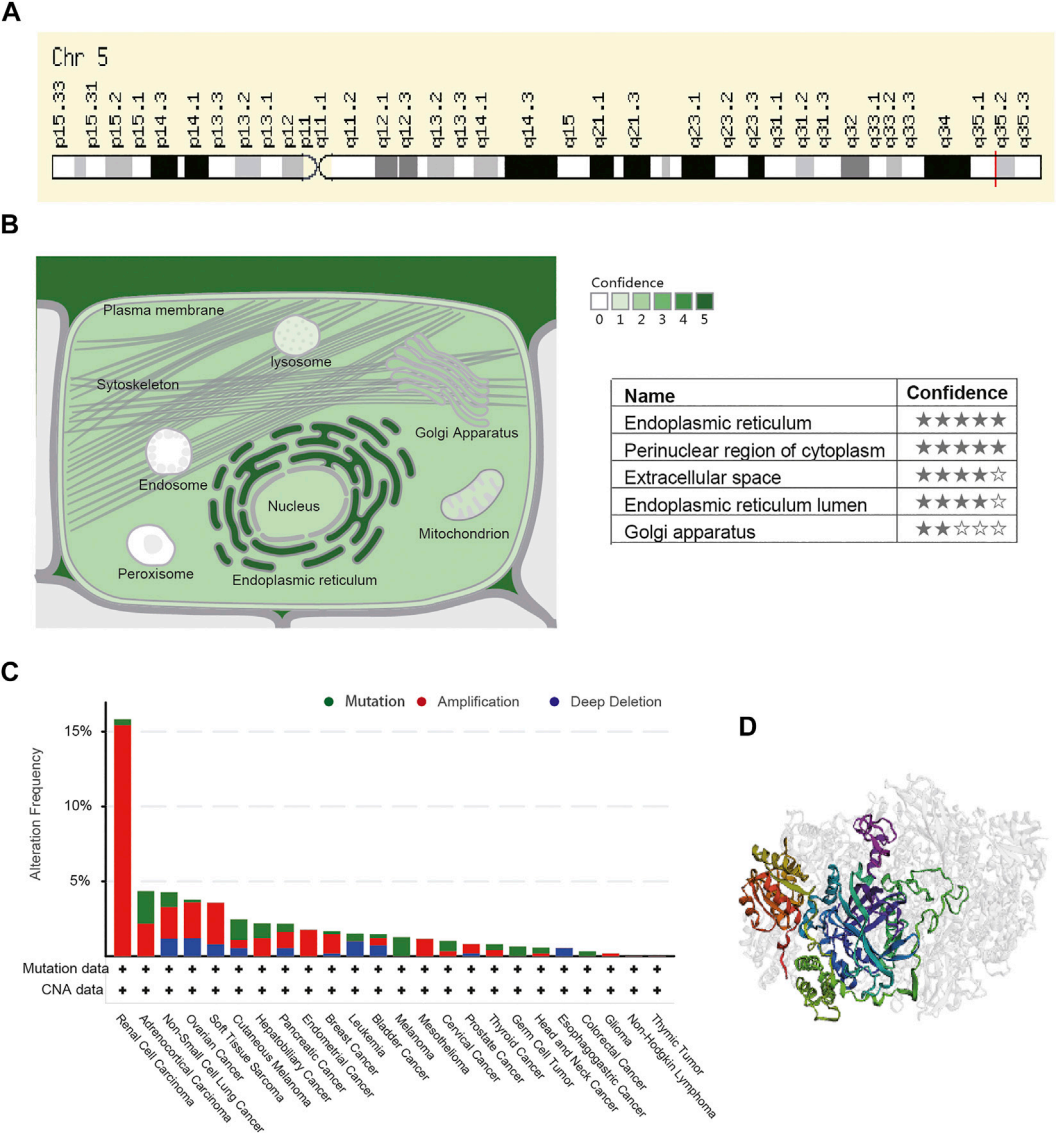
Basic biology of STC2 and genetic alteration analysis. **(A)** Genomic location of STC2 gene in the GeneCards website. **(B)** Subcellular location of STC2 by COMPARTMENTS. **(C)** Mutation type and frequency of STC2. **(D)** Three-dimensional (3D) structures of STC2.

STC2 is a protein-coding RNA located at q35.2 in Chromosome 5 (Figure 10A). The subcellular location of STC2 was found using COMPARTMENTS. The highest confidence in the subcellular location was in the endoplasmic reticulum and perinuclear region of the cytoplasm (Figure 10B). The cBioportal was used to explore the genetic alteration of STC2. The results revealed 200 mutations of STC2 in 8,681 patients with different tumors. Among them, renal cell carcinoma patients had the highest frequency of STC2 gene alteration (15.82%) and the most alterations were amplification (15.44%, 82/531). The mutation frequency of adrenocortical carcinoma is the highest at about 2.17% (Figure 10C). We present the three-dimensional (3D) structure of the STC2 protein in Figure 10D.

## Discussion

STC2 as a glycoprotein is expressed in the broad-spectrum of human tumor cells and tissues, including breast, bone, esophagus, liver, lung, kidney, ovary, prostate, and stomach (Qie and Sang 2022). Under stress conditions such as hypoxia (Law et al., 2008; Law and Wong 2010), ER stress (Joshi et al., 2015), and nutrient deprivation (He et al., 2019), both transcriptional and post transcription of STC2 were regulated, and in turn drives tumor cell growth, proliferation, and tumorigenesis. Moreover, STC2 is identified as an immune response–related gene and upregulated in T-cells with Th2 response (Jansen et al., 2015; Roche et al., 2018). Importantly, STC2 mediates resistance to chemo- and radio-therapies and promotes the development of tumor cell–acquired resistance (Garnett et al., 2004). Hence, we investigated the correlations of STC2 expression level with prognosis, immune checkpoint, MMRs, TMB, MSI, function notation, and related pathways in 33 distinct tumors based on the data from TCGA and GTEx.

First, we assessed the mRNA expression levels of STC2 in tumors. The results revealed that the expression level of STC2 was significantly up-regulated in most cancers. Furthermore, we investigated the clinical prognostic value of STC2 expression levels in tumors by univariate Cox regression. In line with previous reports, high expression of STC2 suggested an unfavorable prognosis in BLCA, COAD, ESCA, HNSC, KIRP, LIHC, LUAD, MESO, SARC, and THYM. In the progression of COAD, STC2 contributed to cancer cell metastasis by promoting epithelial–mesenchymal transition (Chen et al., 2016). Moreover, STC2 also plays a critical role in the progression of HNSCC. STC2 enhanced PI3K/Akt signaling pathways by upregulation of Snail-mediated decrease of E-cadherin, and promoted HNSCC metastasis (Yang et al., 2017). The overexpression of STC2 leads to poor prognosis in LIHC. STC2 could regulate drug efflux and drug resistance of tumor cells by regulating the expression levels of P-glycoprotein and Bcl-2 protein (Shi et al., 2020). In contrast, favorable prognostic indications were found in BRCA and LGG.

As integral components of the tumor immune microenvironment, tumor-infiltrating immune cells (TIICs) were biomarkers for prognosis and response to immunotherapy in various carcinomas (Zhang and Zhang 2020). Many markers including immune checkpoints, TMB, and MSI have been used to evaluate immunotherapy susceptibility (Vrána et al., 2018; Li et al., 2020). Therefore, we investigated the relationship between STC2 expression level and TIICs, immune scores, and stromal scores in multiple malignancies. We observed a significant positive correlation between STC2 expression level and infiltration levels of B cells, CD4^+^ T cells, CD8^+^ T cells, neutrophil, macrophage, and dendritic cells in PRAD, LIHC, KICH, and KIRC. STC2 was an interaction partner of histidine-rich glycoprotein (HRG) located on the surface of inflammatory cells. HRG was a cation- and heparin-binding, 75 kDa plasma glycoprotein produced by liver hepatocytes (Poon et al., 2011), which could generate antitumor immune responses by controlling the phenotypic switch of macrophages. STC2 could form a histidine-rich glycoprotein/stanniocalcin-2 (HRG/STC2) complex with HRG to regulate murine glioma growth *via* modulation of anti-tumor immunity (Roche et al., 2018). Moreover, the previous animal model study showed that STC2 knockdown in murine mesenchymal stem cells significantly impairs its effect in reducing TNF-α– and IFN-γ–producing CD8^+^ T cells (Chen et al., 2018). It is well known that T cells recognize tumor antigens and delivering them to T-cell receptors is a major process in tumor immune regulation (Grinberg-Bleyer et al., 2017). When T-cell function is impaired, cancer cells can evade immune surveillance by multiple mechanisms. Among them, cancer cells exploit various intrinsic immune checkpoints of T cells to escape from immune surveillance; thus, they are important pathways for escaping (Tan et al., 2017). The immune checkpoints are regulators of the immune system and can attenuate T-cell responses to maintain immune homeostasis and prevent autoimmunity (Liu et al., 2020). However, tumors can use various mechanisms to hijack these pathways to evade immune recognition against tumor antigen-specific T cells (Cohen et al., 2016). Therefore, the expression levels of immune checkpoint genes and their ligands are often up-regulated in the tumor immune microenvironment (Zhao et al., 2018). A significant positive correlation between the expression level of STC2 and the expression levels of immune checkpoint genes was observed in most cancers in our study. These findings suggested that STC2 may play an important role in tumor immune avoidance mechanisms. However, the *in vivo* biological importance of STC2 remains to be further dissected, which will be a target for our future study.

MMRs are key players in a post-replication repair system and are involved in repairing DNA base mismatches and indels during the S phase. As a safety and security system, MMRs play a crucial role in ensuring the stability of DNA replication and avoiding the generation of genetic mutations. Mutations or defects in the MMR genes could contribute to the accumulation of genetic errors, resulting in the occurrence of tumors (Geisler et al., 2003). Therefore, we assessed in detail the association of five MMRs (MLH1, MSH2, MSH6, PMS2, and EPCAM) with STC2 expression levels in 33 cancers. The results of Pearson analysis confirmed that STC2 expression level was significantly and positively correlated with DNA repair genes in most types of cancer. MSI is another marker of genomic instability and is a hallmark of MMR defects (Law and Wong 2010). MSI is closely linked to the occurrence, development, and prognosis of various cancers. A study by Gryfe et al. (2000) shows that high-frequency MSI is an independent predictor of clinical characteristics and prognosis in COAD. Compared with low levels of MSI, high levels of MSI exhibit a better anti-tumor immune response, the ability to inhibit tumor cell growth, improved prognosis, and microsatellite stability (Kim et al., 2016; Marrelli et al., 2016; Mohan et al., 2016). TMB might influence the utility of immunotherapy by affecting the generation of immunogenic peptides. Previous studies have revealed that TMB improves the efficacy of immunotherapy in colorectal cancer (Lee et al., 2019) and non-small cell lung cancer (Devarakonda et al., 2018). Therefore, we investigated the correlation between STC2 expression levels with TMB and MSI in 33 tumors. An important finding was that STC2 expression was significantly associated with TMB in 15 cancers and MSI in five cancers. The results suggest that STC2 might serve as a promising tumor therapeutic target to improve the immunotherapy response.

The lack of tumor specificity and drug resistance remains a significant barrier to effective cancer therapy and might be associated with tumor recurrence. Previous studies indicate that STC2 is upregulated upon exposure to several chemotherapeutic treatments. Miyazaki et al. (2014) found that STC2 was the most highly upregulated gene in anti-VEGF antibody–treated colon cancer. This indicated that STC2 might contribute to the development of acquired drug resistance to anti-VEGF treatment. Wang et al. (2015) found that STC2 could promote cell proliferation and cisplatin resistance in cervical cancer by regulating the activity of the MAPK signaling pathway. STC2 promoted activation of the PI3K–Akt signaling pathway and induced the expression of P-glycoprotein which also increases the drug resistance of tumor cells (Yuan et al., 2016). P-glycoprotein was an efflux pump involved in the absorption, distribution, and excretion of chemical compounds and implicated in the poor penetration of many drugs (Brown et al., 2018). The drug sensitivity analysis based on the CellMiner database found that STC2 expression levels are significantly associated with some drug sensitivity, such as PF-4942847, MPC-3100, SNX-5422, TAK-632, TAS-116, BGB-283, geldanamycin analog, and alvespimycin. It is likely that STC2 upregulated might play an important role in drug resistance.

Genetic alteration is a main factor that drives cancer, including altering genetic content, disrupting genes, and causing phenotypic differences. Cancer genomes typically develop four to five driver mutations when combining coding and noncoding genomic elements, and their instability is a molecular genetic hallmark of tumorigenesis (Bray et al., 2018). Gene amplification is a major manifestation of genomic instability and plays a crucial role in various cancers. In recent years, growing evidence has indicated that mutated-gene–targeted therapy is a promising therapy for tumors (Synnott et al., 2017; Kaur et al., 2018). Our study found that STC2 is mutated in most cancers, and the frequency of STC2 gene mutation in renal cell carcinoma patients reached 15.2%; most of the alterations were amplification. This result further demonstrates that STC2 is a plausible therapeutic target.

To our knowledge, this study is the first comprehensive pan-cancer analysis of STC2. This study systematically investigated pan-cancer information from multiple databases. However, there are still inevitable limitations in our study. First, we did not find that STC2 was differentially expressed between some tumor tissue (BLCA, BRCA, and ESCA) and normal tissue in the previous results of analysis of differentially expressed genes. Therefore, the diagnostic and prognostic value of STC2 in BLCA, BRCA, and ESCA should be further validated with a large number of cases. Second, as our study is limited to the analysis of the existing data, more experimental studies are needed to validate these bioinformatics results. Further research will contribute to clarifying the role of STC2 at the molecular level. Third, although pan-cancer analysis results support that STC2 expression was associated with immunomodulatory mechanisms and immune cell infiltration, the underlying mechanism remains to be further investigated.

## Conclusion

In conclusion, we analyzed data in public databases and found the abnormal expression of STC2 is significantly correlated with the prognosis of HNSCC and can be used as a biomarker for the prognosis of HNSCC. In pan-cancer analysis, STC2 is significantly associated with prognosis, immune cell infiltration, immune checkpoints, immune scoring, mechanistic scoring, TBM, MSI, and MMRs in multiple cancers. This provides a certain basis for bioinformatics analysis for future studies of STC2 in tumor immunity and tumor microenvironment. However, the aforementioned results still require further biological experiments to verify the function and molecular mechanism.

## Glossary

HNSCC: Head and neck squamous cell carcinoma
HPV: Human-papillomavirus
STC2: Stanniocalcin-2
DEGs: Differentially expressed genes
ICG: Immune checkpoint gene
TMB: Tumor mutation burden
MSI: Microsatellite instability
MMRs: Mismatch repair genes
TCGA: The Cancer Genome Atlas
GTEx: Genotype-tissue expression
KEGG: Kyoto Encyclopedia of Genes and Genomes
GO: Gene Ontology
ROC: Receiver operating characteristics
AUC: Area under the curve
HPA: Human Protein Atlas
OS: Overall survival
DFS: Disease-free survival
DSS: Disease-specific survival
PFS: Progress-free survival
HRG: Histidine-rich glycoprotein
HRG/STC2: Histidine-rich glycoprotein/stanniocalcin-2
ACC: Adrenocortical carcinoma
BLCA: Bladder urothelial carcinoma
BRCA: Breast invasive carcinoma
CESC: Cervical squamous cell carcinoma and endocervical adenocarcinoma
CHOL: Cholangiocarcinoma
COAD: Colon adenocarcinoma
DLBC: Lymphoid neoplasm diffuse large B-cell lymphoma
ESCA: Esophageal carcinoma
GBM: Glioblastoma multiforme
HNSC: Head and neck squamous cell carcinoma
KICH: Kidney chromophobe
KIRC: Kidney renal clear cell carcinoma
KIRP: Kidney renal papillary cell carcinoma
LAML: Acute myeloid leukemia
LGG: Brain lower grade glioma
LIHC: Liver hepatocellular carcinoma
LUAD: Lung adenocarcinoma
LUSC: Lung squamous cell carcinoma
MESO: Mesothelioma
OV: Ovarian serous cystadenocarcinoma
PAAD: Pancreatic adenocarcinoma
PCPG: Pheochromocytoma and paraganglioma
PRAD: Prostate adenocarcinoma
READ: Rectum adenocarcinoma
SARC: Sarcoma
SKCM: Skin cutaneous melanoma
STAD: Stomach adenocarcinoma
TGCT: Testicular germ cell tumors
THCA: Thyroid carcinoma
THYM: Thymoma
UCEC: Uterine corpus endometrial carcinoma
UCS: Uterine carcinosarcoma
UVM: Uveal melanoma
