# Supplementary figures and images for: A Pan-Cancer Analysis Reveals the Prognostic and Immunotherapeutic Value of Stanniocalcin-2 (STC2)
Source: Front Genet. 2022 Jul 22;13:927046. doi: 10.3389/fgene.2022.927046 (PMC9354991; doi:10.3389/fgene.2022.927046)

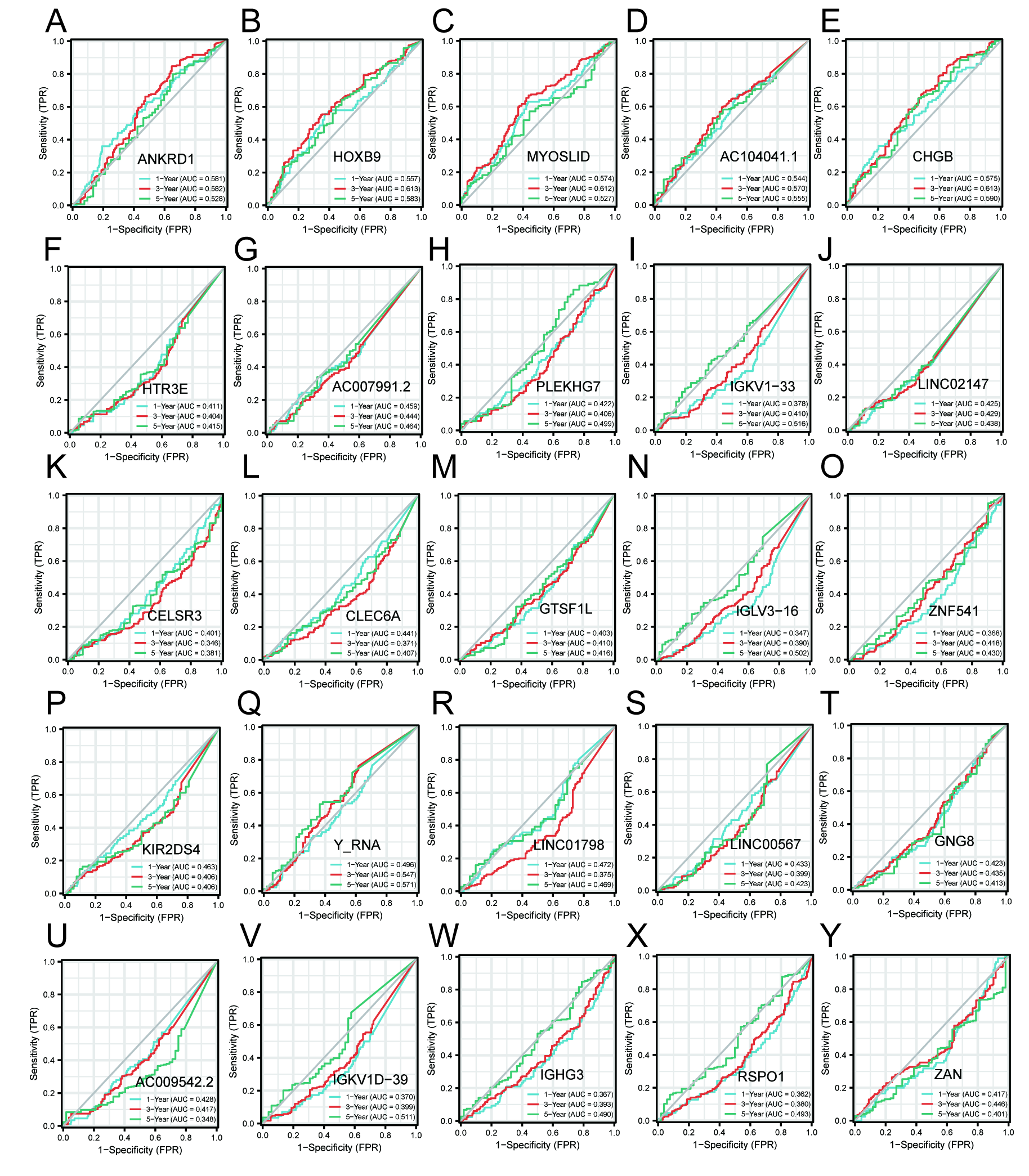

Supplement: Supplementary file 1 [file Image1.TIF]
